# Cross-Brain Neurofeedback: Scientific Concept and Experimental Platform

**DOI:** 10.1371/journal.pone.0064590

**Published:** 2013-05-17

**Authors:** Lian Duan, Wei-Jie Liu, Rui-Na Dai, Rui Li, Chun-Ming Lu, Yu-Xia Huang, Chao-Zhe Zhu

**Affiliations:** State Key Laboratory of Cognitive Neuroscience and Learning, Beijing Normal University, Beijing, P. R. China; Tokai University, Japan

## Abstract

The present study described a new type of multi-person neurofeedback with the neural synchronization between two participants as the direct regulating target, termed as “cross-brain neurofeedback.” As a first step to implement this concept, an experimental platform was built on the basis of functional near-infrared spectroscopy, and was validated with a two-person neurofeedback experiment. This novel concept as well as the experimental platform established a framework for investigation of the relationship between multiple participants' cross-brain neural synchronization and their social behaviors, which could provide new insight into the neural substrate of human social interactions.

## Introduction

Human social cognition is an attractive and important topic in cognitive neuroscience [1]. In recent years, simultaneously measuring multiple brains using electroencephalograph (EEG), functional magnetic resonance imaging (fMRI) or functional near-infrared spectroscopy (fNIRS), together termed hyperscanning, has been developed to explore the neural substrate of social interaction [2]–[4]. Using this novel technology, cross-brain neural synchronization has been found between participants during various social interactions such as body movement coordination and imitation [5]–[7], reciprocal exchange [8], [9], cooperative button-pressing [4] and face-to-face communication [10]. These interesting results suggest that cross-brain neural synchronization exists widely and may play an important role in human social interaction. However, the behavioral significance and the mechanism of this neural synchronization remain vague.

Neurofeedback (NFB) is a promising approach to investigate the relationship between brain activity and behavior. NFB feeds back the neural signatures of a participant to allow him/her to voluntarily regulate his/her own brain activity. Compared with the “behavioral manipulation – brain observation” paradigms in traditional brain imaging studies, NFB enables researchers to manipulate the brain activity as an independent variable and observe the behavioral effect as a dependent variable, which can provide more causal insights into the relationship between brain and behavior (see [11], [12] for a review). Within the past decade, there is converging evidence that a single participant's brain activity can be self-regulated with NFB, yielding specific behavioral effects [11], [12]. Moreover, in 2004, Goebel et. al. for the first time extend NFB from single-person context to multi-person situation (BOLD brain pong, [13], [14]), which is a big step in NFB development. This pioneering work allows multiple participants simultaneously self-regulate their own neural activities in a social interacting situation.

On this foundation, aiming to explore the relationship between the cross-brain neural synchronization and the social behavior, we went a further step to propose a different type of multi-person NFB with the neural synchronization between two participants as the direct regulating target, termed as “cross-brain NFB”. This new concept attempts to extend the regulating target from participants' own neural activities to their cross-brain neural synchronization, which may provide a new researching paradigm for social cognitive neuroscience studies.

There are several steps to implement the cross-brain NFB concept. First of all, an experimental platform is needed to measure multiple participants' neural activities simultaneously, on-line calculate and feed back the neural synchronization information. As a second step, one can use the platform to conduct cross-brain NFB experiments to make two participants regulate their cross-brain neural synchronization of interest. For example, to study social motor such as body movement coordination [5], the correlation between the neural activities of the motor areas of the two brains may be chosen as the regulating target. Directed by the feedback of the cross-brain correlation calculated on-line, participants can try various mental strategies such as kinesthetic motor imagery (i.e. imagine the feeling that actual task performance produces) to regulate the cross-brain correlation together with their partner. Finally, after the participants have learned how to regulate the cross-brain correlation between their motor areas, the behavioral effects of the regulation (e.g. change of the finger and/or foot movement synchrony between pre- and post- regulation) can be investigated between experimental group and control group. Moreover, long-term cross-brain NFB training can be conducted to investigate whether cross-brain NFB training could induce social functional brain reorganization and social behavioral changes, like the single-person NFB training [11].

As a first step, the present study aims to establish an experimental platform for further cross-brain NFB studies. To choose the brain imaging modality for the platform, several demands are considered. Firstly, this imaging modality should be comfortable for participants to conduct frequent and long-term cross-brain NFB experiments. Secondly, it should have good ecological validity to provide a naturalistic environment for social interaction. Thirdly, it should be low-cost for use and maintenance. Among the various noninvasive brain imaging modalities, EEG exhibits the advantages of low-cost, portability, and the ability to simultaneously measure multiple participants. However, EEG restricts the motions of the participant's body and eyes, and requires an injection of the conducting gel, which may decrease the comfort of the participants. fMRI has excellent spatial resolution, and has been used in previous multi-person NFB studies [13], [14]. However, fMRI is expensive, uncomfortable, non-portable and sensitive to head motion. It is also a challenge for fMRI to support direct face-to-face communication between two participants.

On the other hand, fNIRS offers a cost-effective non-invasive brain imaging technology to cross-brain NFB. fNIRS is relatively insensitive to the participant's motion and provides a quiet and comfortable scanning environment [15]. fNIRS can easily scan two participants simultaneously with a single fNIRS instrument [4], and the two participants can communicate with each other face-to-face in a natural environment [10]. fNIRS is portable and flexible, which provides further potential to gather multiple fNIRS equipments to conduct social interaction studies on a larger participant group. Accordingly, in the present study, a cross-brain NFB experimental platform was established on the basis of fNIRS, and was validated with a two-person NFB game experiment.

## Experimental Platform

**Figure 1:**
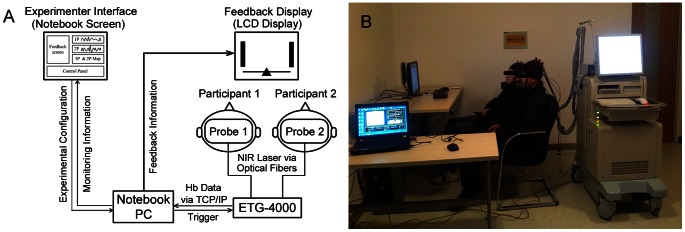
The fNIRS-based experimental platform. (A) The framework of the platform. (B) An overview of the experimental scenario. Both two participants have given their written informed consents, as outlined in the PLOS consent form, to publication of their photograph.

The established fNIRS-based cross-brain NFB experimental platform consists of three modules: (1) data acquisition and transmission; (2) online data processing and feedback and (3) an experimenter interface (Fig. 1A).

### Data acquisition and transmission

In this module, the fNIRS measurement was simultaneously conducted on the two participants using one ETG-4000 multi-channel optical topography system (The Hitachi Medical Corporation, Tokyo, Japan). The near-infrared laser optodes (emitters and detectors) were fixed in an elastic holder and placed on each participant's head, which was reinforced by a nylon swimming cap (see Fig. 1B). The absorptions of near-infrared light at two wavelengths (695 nm and 830 nm) were measured with a sampling rate of 10 Hz and converted into concentration changes in hemoglobin parameters (oxygenated- (HbO) and deoxygenated- (HbR) hemoglobin) automatically by the ETG-4000 system using the modified Beer – Lambert law [16] in real-time. The hemoglobin data were transmitted to the data processing module in real-time using the TCP/IP protocol via the Ethernet LAN.

### Online data processing and feedback

The online data processing and feedback module was programmed with MATLAB (R2010a, The MathWorks Corporation), and performed on a PC notebook (Lenovo Thinkpad L421 with Intel i5-2430M CPU and 2G RAM; Microsoft Windows 7 operating system). This module received real-time hemoglobin data from the ETG-4000, and subsequently calculated the feedback information online. The feedback information was output to a display screen in front of the participants via a video cable (Fig. 1B).

Before experiment, the ways to calculate and present the feedback information need to be determined and programmed according to the regulation target. For example, if the correlation between two participants' motor areas is chosen as the regulating target, the neural signals of the two participants' motor areas are extracted to calculate the correlation using on-line algorithm (e.g. calculate the correlation using a sliding window). And the correlation result is visually fed back to the participants using such as graphical thermometers (the module can also be easily extended to allow auditory feedback). The feedback calculation and presentation are programmed as the MATLAB m-files to embed into this module.

### Experimenter interface

The experimenter used a graphic user interface (GUI) program to set the experimental configuration, trigger the measurement, and monitor the brain activities of the participants. This GUI program was developed with MATLAB. For reasons of mobility, this program was also performed on the notebook PC aforementioned and displayed on the notebook screen (using the dual-display technique, the data processing program running on the same PC could output the feedback to an external LCD display).

**Figure 2:**
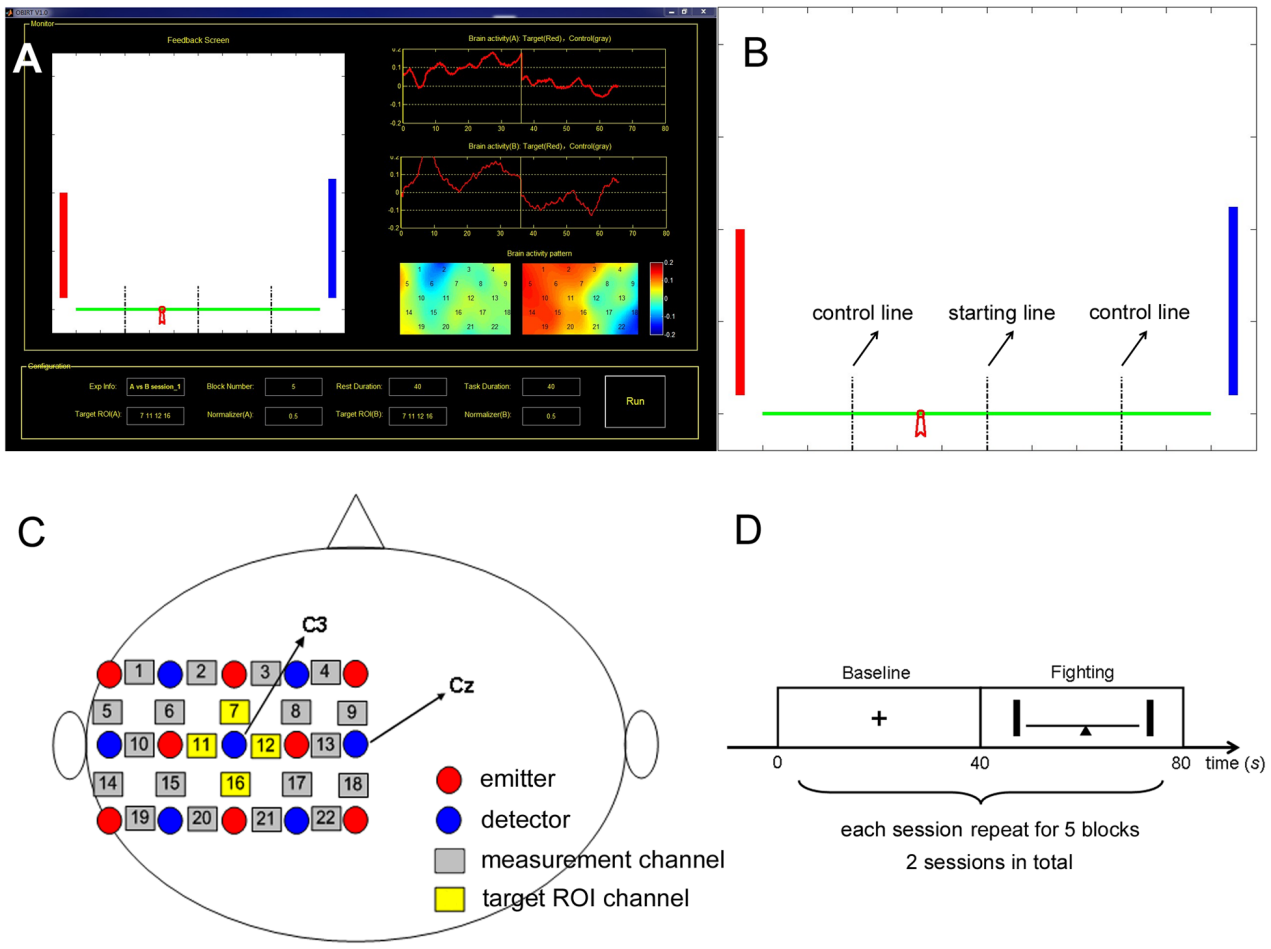
The “tug-of-war” NFB game for platform validation. (A) The experimenter interface. (B) The feedback screen for the participants. The green horizontal line represented the rope, with a red ribbon on it. Both the participants' brain activities in the target ROI were also fed back onto the screen (the red bar and blue bar for 1P and 2P, respectively). (C) Arrangement of the optodes and measurement channels. (D) Paradigm of the experiment.

The GUI provided a parameter configuration panel (the bottom panel in Fig. 2A). The experimental parameters included the number and duration of the blocks, and the target region of interest (ROI) of each participant used to calculate the feedback information. A “Run” button was provided to start the experiment. When the button was pressed, the other two modules (data acquisition and transmission; online data processing and feedback) were triggered synchronously.

The experimenter could monitor the participants' brain activity online, as shown in the top-right panel in Fig. 2A. The two time-course windows separately illustrated each participant's hemoglobin signals in the target ROI. The brains' spatial activity patterns were also displayed in real-time. The refresh rate of the curves and maps was 10 Hz. The top-left panel was a real-time copy of the participants' feedback screen to enable the experimenter to observe the potential relevance between the feedback and brain activities.

## An Experiment for Platform Validation

### The “tug-of-war” NFB game

To test the fNIRS-based multi-person BCI platform, we conducted a demo experiment using a simple “tug-of-war” game. Two participants (denoted as 1P and 2P) fought a cross-brain “tug-of-war” against each other. A rope with a ribbon on it was displayed on the screen (Fig. 2B, the green horizontal line represented the rope). Initially, the ribbon was positioned at the midpoint of the rope (the starting line). The target of both participants was to pull the ribbon back to his side by regulating his brain activity in the left sensorimotor area higher than his opponent using kinesthetic motor imagery. The difference between the amplitudes of their brain activities at each time point corresponded to the amount of the ribbon's shift.

### Participants

Two male right-handed volunteers (age 23 and 25 years) recruited from Beijing Normal University participated in this experiment, with written informed consent obtained. The experimental protocol was approved by the Institutional Review Board at State Key Laboratory of Cognitive Neuroscience and Learning, Beijing Normal University.

### fNIRS measurement

The demo experiment was performed in a silent and dim room. Each of the participants sat in a soft chair. All of the 44 measurement channels were divided into two halves and each participant's brain activity was acquired using one-half. A 3×5 probe set consisting of eight emitters and seven detectors was used on each participant, forming 22 measurement channels (Fig. 2C). The probe set was placed over the participant's left parietal brain to cover the left sensorimotor area in accordance with the international 10–20 system [17]. The detector in middle of the right-most of the probe set was placed on Cz, thereby making the detector in the center of the probe set positioned on C3. The four adjacent channels of the detector on C3 (Channel 7, 11, 12, 16) were selected as the target ROI for feedback regulation [18], [19]. The configurations of the probe set on both of the participants were identical.

### Paradigm

The participants underwent two sessions of games. Each session lasted 7 minutes and 10 seconds (430 s) including an initial 30-s pre-scan time and subsequent 5 blocks of “fighting round” (each block lasted 80 s). The 30-s pre-scan time was used for steady-state control and was discarded in further analysis. Each “fighting round” block consisted of a 40-s baseline and a 40-s mental “fighting” of motor imagery (Fig. 2D). There were 5 minutes between the two sessions for the participants to rest.

The participants were instructed to perform kinesthetic motor imagery during the fighting rounds to fight against his opponent, and to adjust their imagery strategy according to the competing situation. Both participants have accepted the single-person NFB training in performing kinesthetic motor imagery before. In this experiment, they could use any motor imagery strategies they had learned. They were also told to breathe peacefully and remain as motionless as possible during the entire experiment.

### Feedback calculation

In the present study, only the HbO signals were used to calculate the feedback information due to the higher signal-to-noise ratio (SNR) of the HbO compared to the HbR [15]. Specifically, the HbO signals were first moving-averaged with a 10-timepoint sliding window to remove the high frequency noises. For the signals in each fighting round, the mean of the 2-s baseline signals just before the imagery was subtracted channel-wise to reduce the baseline drift. The difference between the average signal amplitudes in the pre-defined target ROI of two participants was used as the feedback information to shift the ribbon on the screen. The averaged signal amplitudes of the two participants were also presented with red and blue bar, respectively.

## Results and Discussions of the Validation Experiment

### On-line neurofeedback regulation

Both participants reported that playing this competing game with another person was very exciting and they can perform the motor imagery better than in single-person NFB. They also reported that because their attentions were highly concentrated, they got fatigued easier than in single-person NFB, especially for the last two blocks of each session.

**Figure 3:**
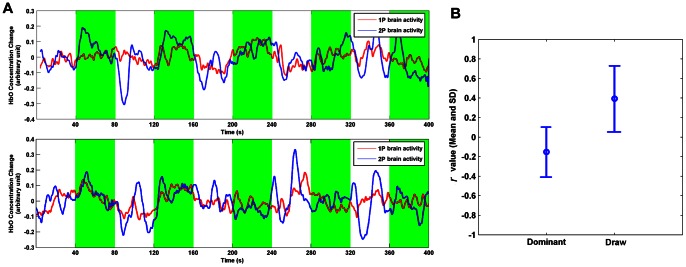
Results of the validation experiment. (A) Data from the two sessions. The upper shows the first session and the lower shows the second session. The red lines and blue lines illustrate the brain activities in the ROI of 1P and 2P, respectively. The fighting time is highlighted in the green color. The raw signals were bandpass filtered (0.01–0.5 Hz) to reduce the low-frequency drifts and high-frequency noises such as thermal noise and pulse rate. (B) The mean and standard error of the *r* values in the draw and dominant blocks, respectively.

The neural data of the two participants from two sessions are shown in Fig. 3A. The red line and blue line represent the brain activities in the target ROI of the 1P and 2P participants, respectively. The fighting time is highlighted in the green color. Both participants have done a good job in regulating their brain activities. 1P player has regulated his brain activity significantly higher than the baseline for block #1, #2, #3, #4 of session 1 and block #1, #2, #3 of session 2. While 2P player has regulated his brain activity significantly higher than the baseline for block #1, #2, #3, #4 of session 1 and block #1, #2, #5 of session 2 (one-tail two-sample *t*-test, threshold *p*<0.05). For both participants, the block number of successful regulation decreased across session (both decreased from 4 blocks to 3 blocks). In addition, within a same session, the early blocks seem to be easier to be regulated than the later ones (For session 1, the two participants only failed to regulate the last block. And for session 2, Participant 1 failed to regulate block #4 and #5 and Participant 2 failed to regulate block #3 and #4). These results showed a fatigue effect of NFB regulation and were consistent with the participants' report.

### Off-line analysis

The present demo experiment used the difference signal between two brains as the feedback information. We have also performed an off-line analysis to make a preliminary attempt to explore the neural synchronization between two participants during different interaction states in the NFB regulation. First, different interaction states between the two participants were characterized according to the fighting results. All the ten rounds from two sessions were post-hoc divided into two categories (“dominant” and “draw”). The rounds in which the ribbon had been pulled back over three-fifths of the half-length of the rope (the control lines in Fig. 2B) and held for more than 10 seconds by either of the two participants was labeled “dominant” (where the victory or defeat was quite clear and there were six rounds in total). In contrast, the other rounds were labeled “draw” (where the victory or defeat was not quite clear and there were four rounds in total). Then, the Pearson correlation coefficient (*r*) was used to measure the cross-brain relationship between the two participants. The *r* values of all the ten rounds were calculated and together compared between the two different fighting results.

As shown in Fig. 3B, for the “dominant” rounds the *r* value was −0.15±0.25 (mean and standard deviation, six samples) and was 0.39±0.33 (mean and standard deviation, four samples) for the “draw” rounds. The *r* values in the “draw” rounds were significantly higher than those in the “dominant” rounds (one-tail two-sample *t*-test, *p*<9×10^−3^).

### Summary

Before we can conduct a real cross-brain NFB experiment which regulates the cross-brain synchronization, the present two-person NFB experiment regulated the difference between two participants' neural activities instead to validate our fNIRS-based experimental platform. The on-line NFB regulation results suggested that both of the two participants could successfully regulate their brain activities in a social interaction situation. The platform was also proved to be competent to measure and integrate two participants' neural activities to calculate the cross-brain feedback information. In addition, the neural synchronization difference between the two fighting conditions found in the off-line analysis was interesting, although it was obviously a quite preliminary result and still needs further investigation.

## Conclusions

The present study proposed the cross-brain neurofeedback concept for the first time and established an experimental platform based on fNIRS. A validation experiment has demonstrated that the platform can measure and integrate two participants' neural activities to calculate and feedback the cross-brain neural interaction. The cross-brain NFB concept, as well as the experimental platform, provides a promising research framework to provide new insight into human social interactions. On the basis of the present work, next step we plan to conduct a cross-brain NFB study to regulate the neural synchronization between two participants' motor systems and investigate its social behavioral effect on motor coordination and imitation.
